# Editorial: A year in review: discussions in developmental endocrinology

**DOI:** 10.3389/fendo.2023.1213095

**Published:** 2023-05-25

**Authors:** Lawrence M. Nelson

**Affiliations:** Digital Women’s Health Initiative, Mary Elizabeth Conover Foundation, Tysons, VA, United States

**Keywords:** estradiol (17ß-estradiol), endocrine disruptors, policy & institutional actions, longevity, women’s health activism, preventative medicine/care/services, cardiovascular disease, hormone replacement estradiol 17 beta

Molecular endocrinology increases our understanding of endocrine diseases and informs the clinical management of complex medical disorders. *Frontiers in Developmental Endocrinology* bridges fundamental molecular insights with broader global public health concerns. Here, leading researchers report the latest scientific insights in endocrine physiology and metabolism. The aim is a deeper understanding of the developmental aspects of clinical disease pathogenesis, diagnosis, and treatment. We are here to integrate concepts from medical investigators, basic scientists, and clinicians to improve global health care and advance health equity.

This Research Topic reports on new insights, challenges, and future perspectives across critical biological issues. For example, we learn about endocrine-disrupting chemicals, regulation of ovarian follicle steroidogenesis, testicular Leydig cell development, and human embryonic development related to pregnancy and neonatal outcomes.

Yan et al. review the effects of endocrine-disrupting chemicals on placental development in humans. Endocrine-disrupting chemicals (EDCs) are environmental compounds interacting with the endocrine system to alter a range of critical biological processes, including immunity, metabolism, organogenesis, reproduction, and behavior. (**Figure 1**) Scientific evidence demonstrates the human health impacts of exposure to EDCs. There are considerable global economic costs to adverse health outcomes induced by EDC exposure (1). There is a need for a multifaceted international program to address the effects of EDCs on human health and to identify, proactively, hazards for effective regulation. The International Agency for Research in Cancer is an excellent model for such a program (2).

**Figure 1:**
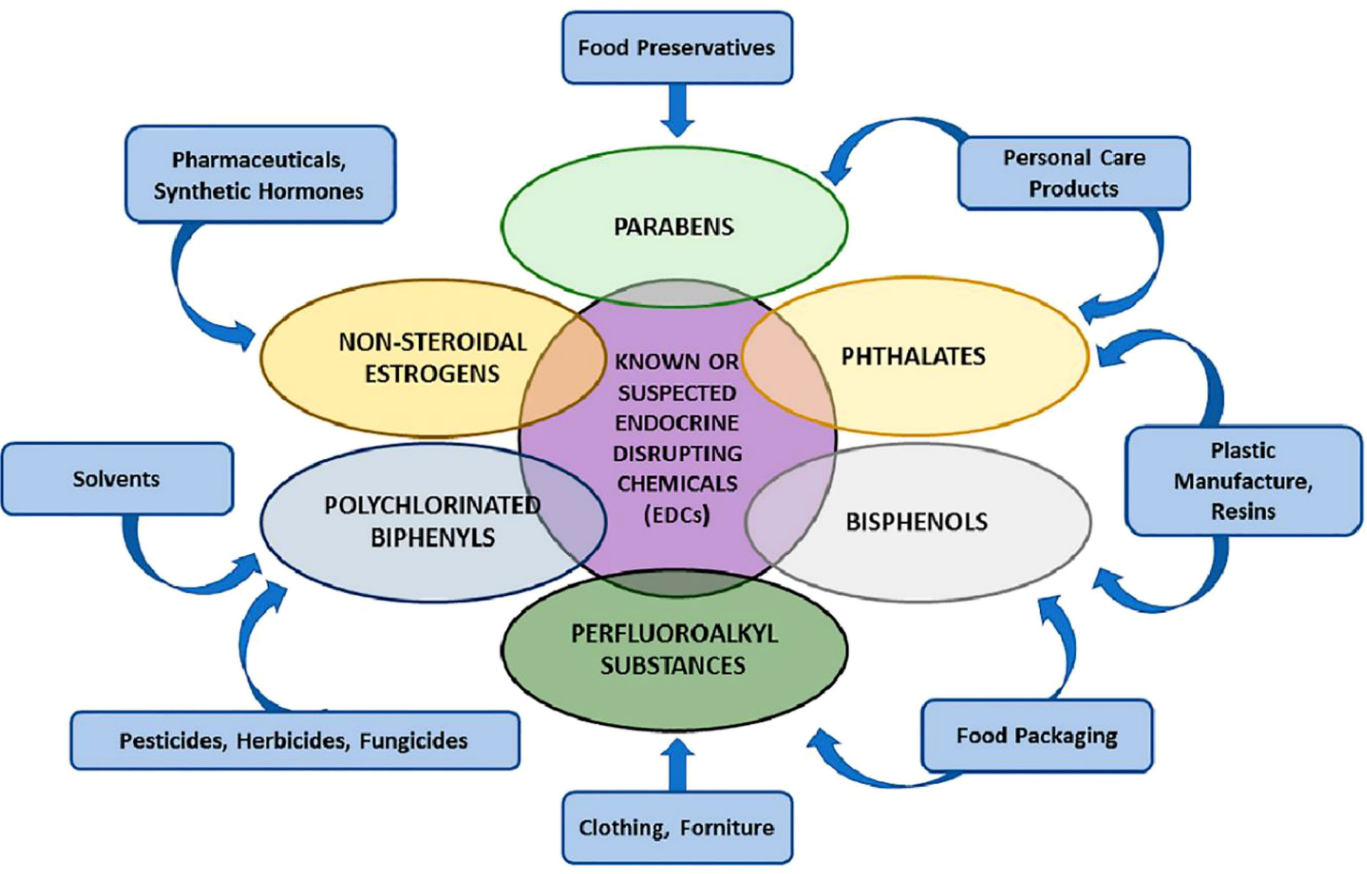
Various groups of endocrine-disrupting chemicals (EDCs) Yan et al.

Devillers et al. investigate molecular aspects of ovarian physiology and examine ovarian follicle estradiol synthesis pathways in mice. Their work provides insight into the expression of several critical intra-ovarian regulators, including the FSH receptor (*Fshr*), the aromatase enzyme (*Cyp19a1)*, and the cell cycle inhibitor p27KIP1 (*Cdkn1b)*.

Estradiol plays an essential role in women’s health across the lifespan. Early onset of estradiol deficiency in women is associated with morbidity and early mortality. These important roles of estradiol include establishing and maintaining bone mineral density and health of the cardiovascular and neurological systems (3–5). The menopause transition to postmenopausal serum estradiol levels is a critical period for cardiovascular health. In women, cardiovascular disease is the most frequent cause of death. Importantly, there is a notable increase in the risk for cardiovascular disease after menopause (6). Intriguingly, arterial stiffness increases within one year of the final menopausal menstrual period (7).

Most estrogen receptors, known as nuclear signaling receptors, regulate gene expression in the cell nucleus. However, a subpopulation of cell membrane estrogen receptors initiates rapid estradiol intracellular signaling, which does not involve transcription, known as “non-nuclear signaling.” The recent development of genetically modified animal models provides new insights into the non-nuclear signaling of estradiol and its role in the cardiovascular system (8).

Women are five years older than men on average when experiencing a first stroke, yet women suffer from more severe strokes (9). A recent systematic review and meta-analysis investigated differences in the presentation of stroke symptoms between the sexes. Women were more likely than men to present with nontraditional symptoms of stroke, a potential factor in delay in diagnosis and treatment. Of note, there was a paucity of studies conducted outside Europe and North America (**Supplementary Figure 1**). The authors called for enhanced equity in global stroke research (9).

There is a critical health need for education and advocacy to put the health benefits of estradiol replacement in proper perspective (10). A recent report in the *New York Times*, “Women have been misled about menopause,” (11) demonstrates that shared decision-making remains a difficult conversation in this area of care (12). Women younger than 45 with estradiol deficiency have a metabolic derangement associated with increased morbidity and mortality (13). Generally, prospective, randomized, double-blind, controlled studies provide the best evidence for making clinical decisions (14). In the past 20 years, the US National Institutes of Health (NIH) Intramural Research Program (NIH-IRP) conducted the only such study on hormone replacement in women with primary ovarian insufficiency (POI) (15). The study provided women with POI with the average daily production rate of estradiol (100 micrograms per day) by transdermal patch and cyclic, monthly oral progestogen. The NIH-IRP hormone replacement regimen restored bone mineral density to normal over three years, and women tolerated the treatment well. Oral estrogen treatment has a higher risk of thromboembolism than transdermal estradiol replacement, yet this more physiologic approach is underutilized in clinical practice (16). Rather than starting from a position of equipoise, future studies of estradiol replacement therapy would best start by considering the benefits of physiologic estradiol replacement by skin patch or vaginal ring compared to oral estrogens.

Bhattacharya et al. publish a review on testicular Leydig cell development in fetal and adult testes. They highlight the cellular progenitor/stemcell origins with associated functional significance in rodents and primates. Recent studies suggest that a small fraction (5-20%) of fetal Leydig cells persist in adult testis. Progress in *ex vivo* cell/organ culture, genome-wide analysis, genetically manipulated mouse models, lineage tracing, and single-cell RNA-seq experiments reveal different steroidogenic outputs of these two populations of Leydig cells.

Chen et al. report on human embryo vacuoles, cytoplasmic inclusions containing liquids from the perivitelline space. Vacuolization in human embryos on Days 3 and 4 are associated with impaired blastocyst development. However, in cases where the rejection of the vacuole-containing cells occurs during the compaction process, blastocysts had a low mosaicism rate. This noteworthy finding supports the hypothesis that excluding abnormal blastomeres during compaction is a self-correction mechanism. Furthermore, the pregnancy rates and neonatal outcomes of vacuole-positive embryos were similar to those of vacuole-negative embryos. Thus, clinicians may consider vacuole-positive embryos an option for embryo transfer.

## Author contributions

The author confirms being the sole contributor of this work and has approved it for publication.
