# Supplementary figures and images for: Editorial: A year in review: discussions in developmental endocrinology
Source: Front Endocrinol (Lausanne). 2023 May 25;14:1213095. doi: 10.3389/fendo.2023.1213095 (PMC10248505; doi:10.3389/fendo.2023.1213095)

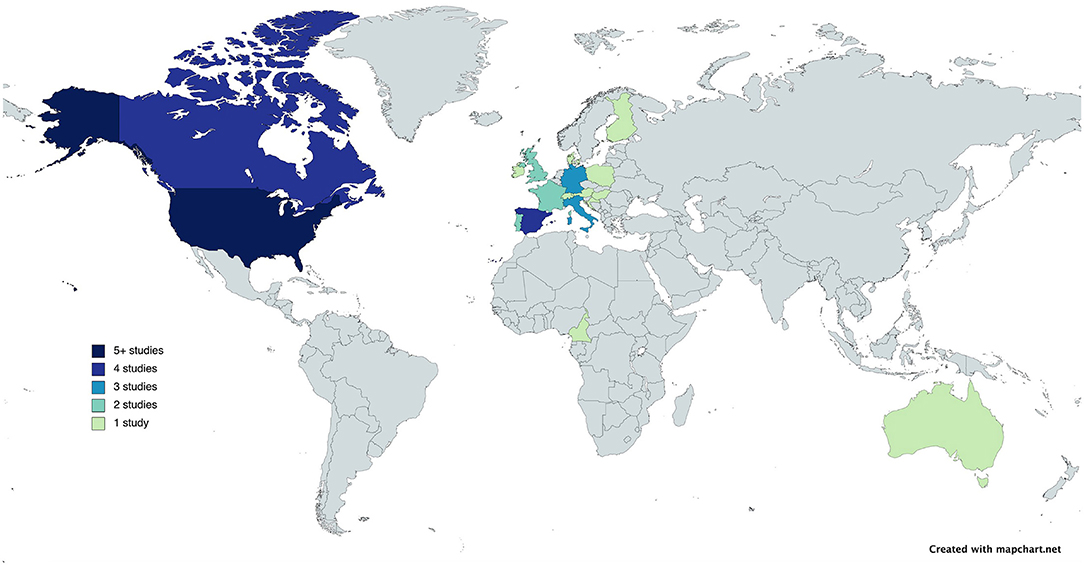

Supplement: Supplementary file 1 [file Image_1.jpeg]
